# Cross-Modal Graph Attention for Bridge SHM Data Imputation

**DOI:** 10.3390/s26113339

**Published:** 2026-05-25

**Authors:** Jiawei Xiong, Liangliang Hu, Xiaolin Meng, Xiangdong An, Yilin Xie

**Affiliations:** 1 State Key Laboratory of Comprehensive PNT Network and Equipment Technology, School of Instrument Science and Engineering, Southeast University, Nanjing 211189, China; 2 The UK-China Intelligent Mobility Research Center, Southeast University, Nanjing 211189, China; 3 College of Architecture and Civil Engineering, Beijing University of Technology, Beijing 100124, China; 4 Jiangsu Hydraulic Research Institute, Nanjing 210098, China

**Keywords:** bridge structural health monitoring, deep learning, missing data imputation, graph attention network, cross-modal attention mechanism, multi-source heterogeneous time series

## Abstract

Bridge structural health monitoring (SHM) systems often suffer from large-scale data missing due to sensor faults, communication interruptions and other reasons during long-term operation, which seriously restricts the reliability of structural state assessment and maintenance decision-making. Compared with conventional single-channel independent modeling strategies commonly used for data imputation, their inherent neglect of spatial correlations and cross-modal causal associations among multi-source heterogeneous monitoring data such as displacement, wind speed, and temperature constrain the imputation capability, particularly when the target channel suffers from long-term continuous data loss. To address the above problems, this paper proposes a collaborative imputation framework integrating a graph attention network (GAT), a modal-aware cross-attention (MACA) mechanism and temporal encoder–decoder architecture (ITimeGAN). Firstly, the sensor feature topological graph is constructed based on the Pearson correlation coefficient, and the spatial dependency among multi-source features is adaptively learned through GAT. Then, the MACA module is introduced, which takes the target displacement as Query and environmental loads as Key/Value, and dynamically aggregates cross-modal driving information through multi-head attention. Finally, a bidirectional LSTM encoder and a unidirectional LSTM decoder are adopted to capture long-range temporal dependencies, so as to realize the accurate reconstruction of missing displacement data. Validated on the 9-dimensional real-world monitoring data from the GeoSHM system of the Forth Road Bridge (UK) under both random missing (10–50%) and continuous long-term missing (1–10 days) scenarios, ITimeGAN achieves an R^2^ of 0.9950 (MAE = 4.25 mm) for longitudinal displacement and 0.9759 (MAE = 6.70 mm) for vertical displacement even under 10 consecutive days of complete data absence. Ablation analysis further reveals that the incorporation of graph attention and cross-modal attention modules reduces the longitudinal displacement MAE by 57% over the baseline, with the imputation performance ranking across three displacement directions being fully consistent with the underlying physical correlation strengths, thereby confirming the effectiveness of the proposed cross-modal collaborative strategy.

## 1. Introduction

Bridges are critical transportation infrastructure whose safety directly impacts public welfare. To monitor structural conditions in real time, countries worldwide have increasingly deployed SHM systems integrating GNSS receivers, strain sensors, and environmental monitoring devices on major bridges [1,2], providing continuous data on displacement, vibration, and stress for condition assessment and maintenance planning [3]. Recent SHM studies also show that high-resolution monitoring technologies, such as optical fiber sensing, have been used in in situ testing and long-term monitoring of civil structures to acquire continuous strain, temperature, and local deformation information, thereby providing an important data basis for interpreting structural behavior [4,5]. In long-span bridge monitoring, GNSS-based remote sensing has also demonstrated outstanding capability; for example, An et al. achieved simultaneous millimeter-level displacement and milli-degree-level attitude determination through an Unscented Kalman Filter framework [6]. However, SHM systems inevitably suffer from data loss during long-term operation due to transmission interference, sensor malfunctions, and harsh environmental conditions, which compromises the reliability of structural assessments and may introduce systematic biases into life-cycle management decisions [7]. Effectively reconstructing missing monitoring data is therefore of great significance for real-time condition evaluation and long-term safety prediction of bridge structures.

To address this problem, data imputation techniques have gradually shifted from traditional model-driven paradigms to data-driven paradigms. Existing methods can be broadly categorized into statistical imputation, shallow learning, and deep learning approaches. Statistical methods, including mean imputation [8], multiple imputation [9], expectation-maximization algorithms [10], and Bayesian inference [11], generally rely on specific distributional assumptions and can perform reasonably well under low missing rates, but they struggle to capture the high-dimensional nonlinear dynamic characteristics of bridge monitoring data. Shallow learning methods such as K-nearest neighbors [12] and maximum likelihood estimation [13] rely on local similarity assumptions and have limited capability in handling structural missingness lasting several hours or longer. Deep learning methods demonstrate greater potential by learning implicit mappings through neural networks. The review by Jia and Li shows that CNN, RNN/LSTM/GRU, AE, GAN, Transformer, and graph neural networks have been applied to SHM tasks such as damage identification, anomaly detection, structural response prediction, and missing data recovery [14]. In specific imputation models, BP neural networks and Generalized Regression Neural Networks have been used for wind field reconstruction [15], while multi-task Gaussian process regression [16], Bayesian dynamic linear models [17], and hybrid frameworks proposed by Chen, Ren, and Li et al. [18,19,20] have also achieved progress in specific monitoring scenarios. Recent self-attention-based imputation models further indicate that simple linear interpolation, PCHIP, or KNN methods are inadequate for high-frequency, nonstationary signals and long-duration continuous missingness; in contrast, SAITS can simultaneously capture long-range temporal dependencies and cross-feature correlations, significantly reducing imputation errors in complex sensor networks while maintaining high computational efficiency [21]. From a broader perspective of intelligent structural engineering, machine learning has also expanded from state identification and performance prediction to inverse design, automated design, and engineering decision optimization, such as inverse design of perforated beams [22] and low-carbon reinforced concrete beam optimization [23]. These studies further indicate that high-quality, continuous, and reliable structural monitoring data are an important foundation for subsequent intelligent assessment, design optimization, and decision-making models.

Graph learning and attention mechanisms provide another pathway for multi-source monitoring data fusion. Niu et al. used a spatiotemporal graph attention network to restore missing SHM data, demonstrating that explicit modeling of sensor relationships can improve reconstruction performance [24]. Ahmad et al. further represented SBAS-InSAR time-series deformation data as a unified spatiotemporal graph and used a dual-stream spatial GAT and temporal GAT structure to impute approximately 48% missing pixels, showing that graph attention can effectively exploit irregular spatial neighborhoods and temporal continuity for deformation-field reconstruction [25]. In broader multimodal perception tasks, graph attention and cross-modal attention have also been used for image-text retrieval, radar micromotion target recognition, and remote-sensing image-text matching, helping to reduce semantic discrepancies between modalities and improve feature fusion [26,27,28]. These studies indicate that graph-structured modeling and cross-modal attention have become important technical routes for multi-source data fusion, but their target tasks, data structures, and physical constraints still differ from bridge SHM sensor data imputation.

As an emerging data-driven paradigm, Generative Adversarial Networks (GANs), since their introduction by Goodfellow [29], have combined the strengths of generative and discriminative models and undergone a series of improvements [30,31]. They can automatically learn latent distributions and temporal features from historical data, thereby offering a new technical pathway for missing data imputation. In structural monitoring, Hu et al. proposed a real-time updating ANN model for long-span bridges and achieved deformation prediction accuracy with R^2^ > 0.87; however, their method assumes data completeness and does not address sensor failure scenarios [32]. Jiang and Hou et al. used GANs to reconstruct short-term acceleration data [33,34], and SegGAN was found to have favorable reconstruction performance but relatively high computational cost [35]. Despite the powerful nonlinear mapping capabilities of GANs, the aforementioned methods still lack explicit modeling of temporal dynamics when handling time-series data imputation.

Time-series Generative Adversarial Network (TimeGAN) further improves the consistency of generated sequences in both statistical properties and dynamic continuity by explicitly capturing temporal dynamics within a generative framework. This architecture has been extended to engineering domains such as renewable energy scenario generation, physically constrained fault diagnosis, and industrial process monitoring, demonstrating its ability to learn complex temporal patterns from historical data [36,37,38,39,40,41,42,43]. However, a critical gap remains between existing TimeGAN variants and the specific demands of bridge SHM: current implementations predominantly operate on univariate or low-dimensional feature spaces without modeling the spatial topology among distributed sensors, and lack cross-modal attention mechanisms to explicitly capture the causal coupling between heterogeneous data sources (e.g., wind speed driving displacement). These limitations become particularly pronounced in bridge SHM, where multi-source monitoring channels are physically interconnected through structural and environmental mechanisms, and the target channel may suffer from prolonged complete data loss that cannot be recovered without leveraging information from correlated sources.

Although previous studies have explored deep learning, graph attention, and multimodal fusion in structural monitoring and related perception tasks from different perspectives, several issues remain insufficiently addressed for multi-source sensor data imputation in bridge SHM: (1) Insufficient use of directional load–response relationships—Existing temporal imputation models mainly emphasize temporal dependencies or generic cross-feature correlations, but they insufficiently characterize the different roles of bridge displacement responses and environmental loads such as wind speed and temperature, as well as the directional load–response dependency between them. (2) Insufficient integration of bridge sensor topology with long-term target-channel missing scenarios—Existing graph attention-based imputation studies are mostly designed for remote-sensing pixels, traffic grids, or general sensor networks, and have not been fully tailored to low-dimensional multi-source heterogeneous channels in bridge monitoring or to scenarios where the target displacement channel is completely missing for a long duration. (3) Remaining gap between TimeGAN and multi-source heterogeneous SHM requirements—TimeGAN and its variants can learn time-series distributions, but when directly applied to bridge SHM, they often lack explicit sensor-topology encoding and cross-modal information retrieval mechanisms, making it difficult to fully exploit environmental load information that remains available during target-channel missing periods.

To address these limitations, this paper proposes a collaborative imputation framework for bridge monitoring data based on an Improved TimeGAN (ITimeGAN). The main contributions include the following:

1. A MACA mechanism that uses displacement as Query and environmental features as Key/Value, enabling directional cross-modal information aggregation via multi-head attention.
2. A GAT encoder that constructs a sensor topology graph from Pearson correlations and learns spatial dependencies among heterogeneous features through attention-weighted neighbor aggregation.
3. A bidirectional LSTM encoder coupled with a unidirectional LSTM decoder to capture long-range temporal dependencies, enhancing the recovery of continuous missing segments.
4. Systematic validation on the 9-dimensional real-world monitoring dataset of Forth Road Bridge in the UK under both random and continuous long-term missing scenarios.

Following this introduction, Section 2 presents the overall framework of ITimeGAN, detailing the design principles and mathematical formulations of three core modules—graph attention encoding, cross-modal attention aggregation, and temporal encoder–decoder; Section 3 describes the Forth Road Bridge GeoSHM monitoring system and experimental data, including data preprocessing, multi-source data correlation analysis, data partitioning and distribution verification, and missing scenario design; Section 4 validates the effectiveness of ITimeGAN through comparative experiments and ablation analysis under random missing and continuous long-term missing scenarios, and discusses the experimental findings; Section 5 concludes the paper and outlines future research directions.

## 2. Methodology

This chapter presents the proposed collaborative imputation method. Since the multi-source heterogeneous monitoring data exhibit significant spatial correlations and causal relationships that existing channel-independent methods fail to exploit, three cascaded modules are designed to progressively extract and fuse information along the spatial, modal, and temporal dimensions.

### 2.1. Graph Construction Based on Feature Correlation and Graph Attention Encoding

**Figure 1:**
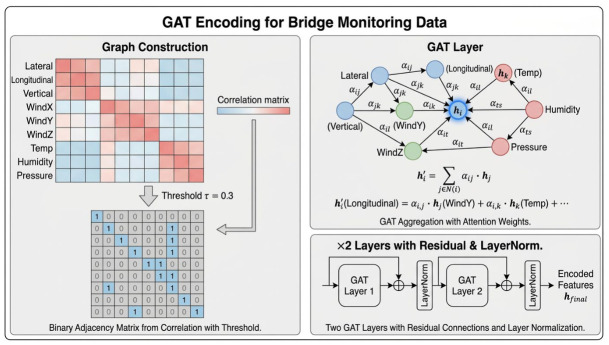
Graph construction from Pearson correlation matrix and GAT-based feature encoding with residual connections.In the correlation matrix, color intensity indicates the Pearson correlation strength, while the different node colors in the GAT layer are used only to distinguish different sensor features.

This section introduces the first module of the framework, the GAT encoder, which aims to explicitly model spatial correlations among multi-source heterogeneous monitoring features, providing the subsequent cross-modal attention computation with correlation-enriched feature representations. The architecture of this module is illustrated in Figure 1.

Graph construction: Given the training dataset $X\in R^{N\times F}$, where N denotes the number of time steps and F the feature dimension, a principled yet computationally efficient metric is required to quantify inter-feature associations for graph edge determination. The Pearson correlation coefficient is adopted for this purpose, as it provides a closed-form, parameter-free measure of linear association strength that is well-suited to the predominantly linear coupling relationships observed between environmental loads and structural responses in bridge monitoring data (e.g., the quasi-linear dependence of longitudinal displacement on wind speed). The pairwise correlation is computed as

(1) $$C_{ij}=\frac{cov(x_{i},\;x_{j})}{\sigma_{x_{i}}\;\sigma_{x_{j}}}$$

where $cov(x_{i},x_{j})$ is the covariance between features $x_{i}$ and $x_{j}$, and σ denotes the standard deviation. To retain only physically meaningful associations, a correlation threshold τ is applied to construct a binary adjacency matrix $A\in R^{F\times F}$:

(2) $$A_{ij}=\left\{\begin{array}{cc} 1, & if\;|C_{ij}|>\tau \;and\;i\neq j\\ 0, & otherwise \end{array}\right.$$

The threshold value is determined through multiple experimental trials to balance the completeness of correlation capture and computational efficiency. The adjacency matrix organizes the F features as graph nodes, with edges representing significant inter-feature associations, as shown in the left panel of Figure 1.

Graph attention layer: GAT is adopted for adaptive learning of feature correlations. For each feature node, a linear transformation first projects it into a d dimensional embedding space, $h_{i}=Wx_{i}$, where $W\in R^{d\times F_{in}}$. The attention score between node i and its neighbor j is then computed as

(3) $$e_{ij}=LeakyReLU\left(a^{⊤}[h_{i}\|h_{j}]\right)$$

where $a\in R^{2d}$ is a learnable attention vector and ‖ denotes concatenation. After masking based on the adjacency matrix A, softmax normalization yields the attention weights:

(4) $$\alpha_{ij}=\frac{\exp(e_{ij})}{\sum_{k\in N_{i}}\exp(e_{ik})}$$

The enhanced representation of node i is obtained by weighted aggregation over its neighbors:

(5) $$h{’}_{i}=\sum_{j\in N_{i}}\alpha_{ij}\;h_{j}$$

Compared with fixed-weight approaches such as GCN, the key advantage of GAT lies in its attention weights $\;\alpha_{ij}$, which assign differentiated importance to different neighbors, thereby more precisely capturing the contributions of critical features—for instance, automatically amplifying the attention weight from wind speed nodes to displacement nodes during strong wind conditions, as depicted in the upper-right panel of Figure 1.

Multi-layer encoder: Two GAT layers are stacked to form the encoder, each equipped with Layer Normalization and residual connections (see the lower-right panel of Figure 1). Both the input and hidden dimensions are set to d=64. After two rounds of feature propagation, the output representation at each time step t, denoted as $H^{\left(t\right)}\in R^{F\times d}$, retains the original information of each feature while incorporating spatial correlation information from related neighbors.

### 2.2. Modality-Aware Cross-Attention Mechanism

**Figure 2:**
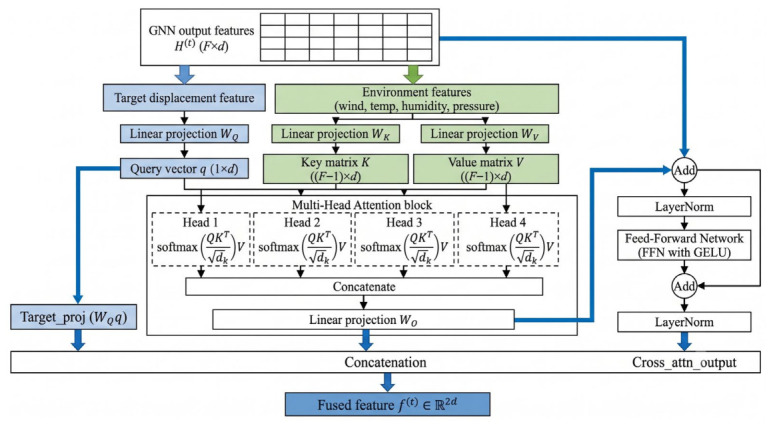
Detailed architecture of the Modality-Aware Cross-Attention (MACA) module with multi-head attention and residual feed-forward network.

The feature representations output by the GNN encoder have captured spatial correlations, but treat all features symmetrically without distinguishing the roles of target variables (bridge displacement) and environmental driving factors (wind speed, temperature, etc.). From a physical perspective, however, displacement is a structural response while environmental parameters are driving inputs, and there exists a clear causal direction between them. This section introduces the MACA mechanism, which uses the target feature as Query to actively retrieve key information from the environmental features, thereby achieving directional information aggregation. The detailed architecture of the MACA module is illustrated in Figure 2.

Query–Key–Value separation: From the GNN output $H^{\left(t\right)}\in R^{F\times d}$, the target feature and environmental features are separated according to their physical meanings. The target displacement feature is linearly projected to generate the Query vector. Unlike standard self-attention, which symmetrically mixes all channels, this design restricts the information flow to target response retrieving environmental drivers, thereby preventing environmental channels from directly dominating the displacement representation without target constraints:

(6) $$q^{\left(t\right)}=W_{Q}h_{target}^{\left(t\right)}\in R^{d}$$

The environmental features (wind speed, temperature, humidity, atmospheric pressure, etc.) are projected to generate the Key and Value matrices:

(7) $$K^{\left(t\right)}=W_{K}H_{env}^{\left(t\right)}\in R^{(F-1)\times d},\;V^{\left(t\right)}=W_{V}H_{env}^{\left(t\right)}\in R^{(F-1)\times d}$$

Multi-head attention computation: To capture cross-modal associations from different subspaces, $n_{head}=4$ parallel attention heads are employed. Each attention head k independently performs scaled dot-product attention in a $d/n_{head}-dimensional$ subspace. If an environmental sensor contains abnormal noise, its consistency with the target Query is usually reduced, and therefore its contribution can be weakened during softmax attention weighting. Together with the preceding outlier preprocessing, this mechanism helps reduce noise leakage from faulty environmental channels into target displacement imputation:

(8) $${Attention}_{k}=softmax\left(\frac{Q_{k}K_{k}^{T}}{\sqrt{d/n_{head}}}\right)V_{k}$$

The outputs of all heads are concatenated and linearly projected: $MultiHead=W_{O}[{Attention}_{1};\ldots ;{Attention}_{4}].$

Residual connection and feed-forward network: The cross-attention output is processed through residual connections and Layer Normalization, followed by a two-layer Feed-Forward Network (FFN) to further enhance the representational capacity:

(9) $$z^{\left(t\right)}=LN(q^{\left(t\right)}+MultiHead(q^{\left(t\right)},K^{\left(t\right)},V^{\left(t\right)}))\;$$

(10) $$z{’}^{\left(t\right)}=LN(z^{\left(t\right)}+FFN(z^{\left(t\right)}))\;$$

where the FFN consists of two fully connected layers with GELU activation, and the intermediate dimension is  2d.

Fused feature construction: The projected original target feature and the cross-attention enhanced feature are concatenated to form a fused feature that encompasses both self-information and cross-modal correlation information:

(11) $$f^{\left(t\right)}=[W_{proj}h_{target}^{\left(t\right)}\|z{’}^{\left(t\right)}]\in R^{2d}$$

This design ensures that even when the cross-attention module fails to effectively aggregate environmental information during certain periods (e.g., when environmental sensors suffer from simultaneous data loss), the intrinsic information of the target feature is still fully preserved rather than being completely overwritten by the cross-modal branch.

### 2.3. Temporal Encoder–Decoder

**Figure 3:**
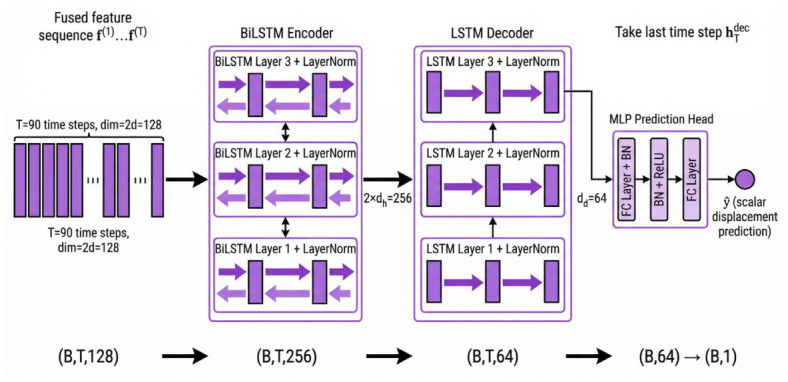
Architecture of the temporal encoder–decoder with BiLSTM encoder, LSTM decoder, and MLP prediction head. In the BiLSTM encoder, the dark purple arrows indicate the forward temporal information flow, while the light purple arrows indicate the backward temporal information flow.

The fused feature sequence $\left\{f^{\left(1\right)},\ldots ,f^{\left(T\right)}\right\}$ has encoded spatial correlations and cross-modal information, but the dynamic dependencies along the temporal dimension have not yet been modeled. Bridge displacement data exhibits significant temporal characteristics: wind-induced displacement fluctuations present sustained trends, while temperature-induced displacement responses exhibit notable lag effects. Drawing on the idea of temporal generative modeling in TimeGAN, this section employs a Bidirectional LSTM encoder and a unidirectional LSTM decoder to form the temporal processing module. The architecture of this module is shown in Figure 3.

Bidirectional LSTM encoder: The fused feature sequence $f\in R^{T\times 2d}$ (T=90, corresponding to a 90-min window) is fed into a 3-layer bidirectional LSTM to extract temporal dependencies. The forward LSTM captures historically accumulated information, while the backward LSTM captures future contextual information. The hidden states from both directions are concatenated to yield the temporal encoding:

(12) $$h_{t}^{enc}\;=\;[\vec{LSTM}(f^{\left(t\right)});\;\overset{\leftarrow }{LSTM}(f^{\left(t\right)})]\in R^{2d_{h}}$$

where the hidden dimension per LSTM layer is $d_{h}=128$, resulting in a concatenated dimension of 256. Layer Normalization is applied after each layer to stabilize deep training.

Unidirectional LSTM decoder: The encoder output sequence is decoded through a 3-layer unidirectional LSTM with hidden dimension $d_{d}=64$, compressing the high-dimensional temporal representation into a compact prediction-oriented feature:

(13) $$h_{t}^{dec}=LSTM(h_{t}^{enc},h_{t-1}^{dec})\in R^{d_{d}}$$

The hidden state at the last time step $h_{T}^{dec}$ is extracted as the temporal feature summary of the entire sequence.

Prediction head: A two-layer fully connected network maps the temporal feature to a scalar prediction:

(14) $$\hat{y}=W_{2}\cdot ReLU(BN(W_{1}h_{T}^{dec}+b_{1}))+b_{2}$$

where BN denotes Batch Normalization, $\;W_{1}\in R^{d_{d}\times 2d_{d}}$, and $W_{2}\in R^{2d_{d}\times 1}$.

### 2.4. Training Strategy and Missing Data Imputation Procedure

Loss function: An equally weighted combination of MSE and MAE is adopted as the training objective, where the former penalizes samples with large deviations and the latter ensures the robustness of overall error:

(15) $$L=0.5\cdot MSE(\hat{y},y)+0.5\cdot MAE(\hat{y},y)\;$$

Training stability design: Considering that conventional GAN training may suffer from gradient instability and mode collapse, this study embeds the temporal generative modeling idea of TimeGAN into a supervised imputation framework. The main training objective is constrained by reconstruction errors rather than relying on unconstrained adversarial loss as the sole optimization signal. Meanwhile, residual connections, Layer Normalization, Batch Normalization, and gradient clipping are introduced within the GAT, MACA, and temporal encoder modules to alleviate gradient vanishing or exploding in deep temporal models.

Optimization strategy: The AdamW optimizer is used with an initial learning rate of $10^{-3}$ and a weight decay of $10^{-4}$, combined with a ReduceLROnPlateau learning rate scheduler (patience = 5, decay factor = 0.5) and an early stopping mechanism (patience = 20). The gradient clipping threshold is set to 1.0.

Iterative bidirectional imputation: For continuous long-term missing scenarios, an iterative bidirectional imputation strategy is designed: for each missing position i, a forward window [i−T,i) and a backward window (i,i+T] are constructed separately; predictions are made independently, and then averaged for fusion:

(16) $${\hat{y}}_{i}=\frac{1}{2}({\hat{y}}_{i}^{fwd}+{\hat{y}}_{i}^{bwd})\;$$

In each iteration, the predicted values are filled back into the input sequence and updated. Three rounds of iteration are performed by default to progressively refine the imputation results and reduce accumulated errors.

### 2.5. Overall Data Flow and Module Synergy

**Figure 4:**
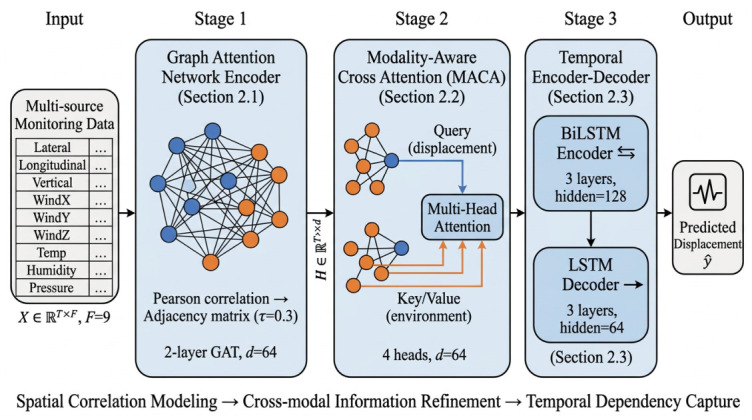
Overall framework of the proposed collaborative imputation method based on GAT-MACA-BiLSTM. In the input table, ellipses indicate time-series values for each monitoring channel. Blue nodes represent bridge displacement response features, and orange nodes represent environmental load features.

As shown in Figure 4, the proposed framework processes the raw 9-dimensional monitoring time series through three cascaded stages. First, the GAT encoder (Section 2.1) maps each feature into a d−dimensional embedding and performs two rounds of attention-weighted neighbor aggregation on the Pearson correlation-based topology graph, producing spatially enhanced representations $H^{\left(t\right)}\in R^{F\times d}$ that encode inter-feature correlations. Next, the MACA module (Section 2.2) separates these representations into target displacement (Query) and environmental (Key/Value) groups, applies multi-head cross-attention to dynamically weight the environmental contributions, and concatenates the result with the projected target feature to yield a fused vector $f^{\left(t\right)}\in R^{2d}$. Finally, the temporal encoder–decoder (Section 2.3) feeds the fused sequence into a 3-layer BiLSTM encoder and a 3-layer LSTM decoder, extracts the last-step hidden state, and maps it to a scalar displacement prediction $\hat{y}$ via the MLP head.

The three modules are jointly optimized end-to-end under the composite loss defined in Section 2.4. Gradient signals propagate backward through the entire pipeline, allowing each module to co-adapt: the GAT encoder learns to emphasize edges most informative for imputation, the MACA module sharpens its attention on the dominant environmental drivers, and the temporal decoder refines its sequence-level reconstruction. This cascaded design forms a progressive information-processing pipeline—spatial correlation modeling → cross-modal information refinement → temporal dependency capture—in which each module’s output serves as increasingly refined input for the next. For continuous long-term missing scenarios, the framework further employs the iterative bidirectional strategy (Section 2.4), performing three rounds of forward–backward prediction and result averaging to progressively reduce accumulated errors.

## 3. GeoSHM Monitoring System and Experimental Data

### 3.1. Forth Road Bridge and GeoSHM Monitoring System

**Figure 5:**
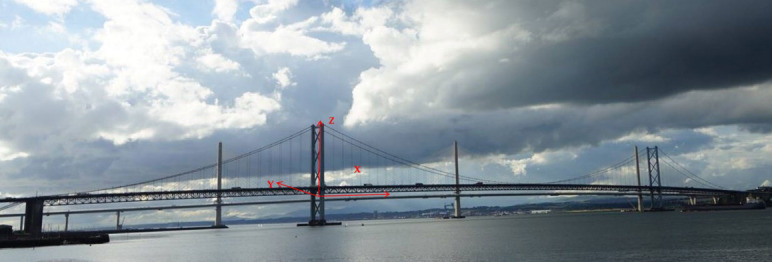
Forth Road Bridge, UK.

The experimental data are obtained from the GeoSHM (GNSS and Earth Observation for Structural Health Monitoring) structural health monitoring system of the Forth Road Bridge (FRB) in the United Kingdom, as shown in Figure 5. FRB is a representative long-span flexible suspension bridge whose structural responses are strongly affected by wind loads and ambient temperature. During long-term monitoring, data loss may also occur due to sensor operating conditions and the marine environment. Therefore, this bridge provides a representative validation scenario for multi-source heterogeneous data imputation methods.

**Figure 6:**
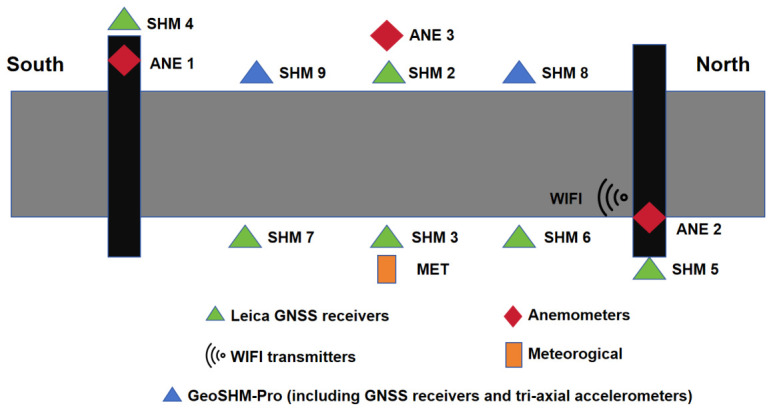
GeoSHM monitoring system of the FRB.

As shown in Figure 6, the GeoSHM monitoring system provides multi-source sensing data for the FRB. In this study, the GNSS three-dimensional displacement at the mid-span SHM2 station, the three-dimensional wind speed at the mid-span, and the temperature, atmospheric pressure, and humidity recorded by the weather station are selected as data sources, forming nine monitoring variables in total. In the bridge coordinate system, the X-, Y-, and Z-directions correspond to lateral, longitudinal, and vertical displacement, respectively.

### 3.2. Dataset Composition and Preprocessing

**Figure 7:**
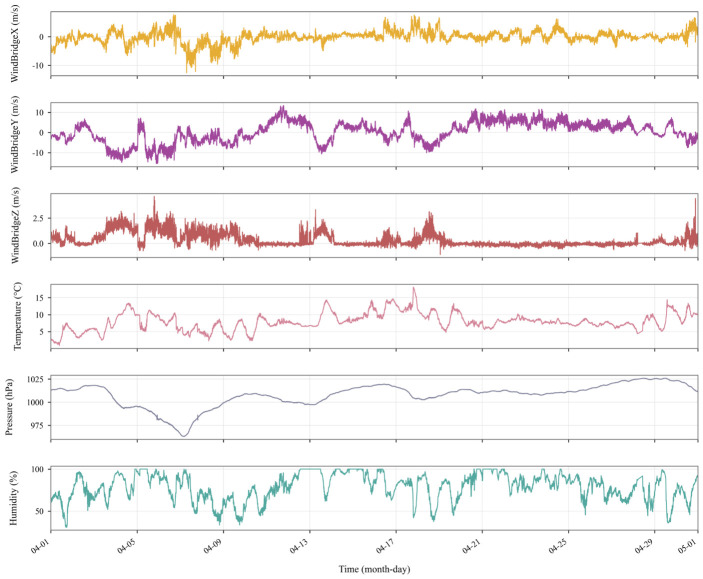
GeoSHM temporal variations in three-dimensional wind speed, temperature, air pressure, and humidity at the SHM2 point in the FRB.

**Figure 8:**
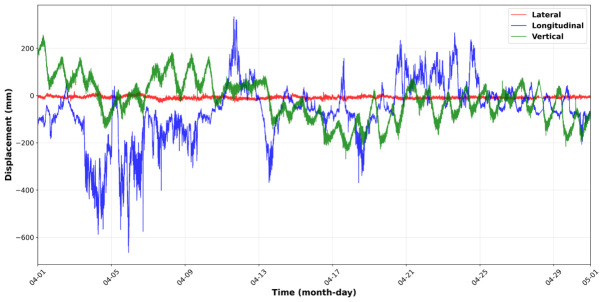
Dynamic deformation at point SHM2 in FRB.

The dataset covers the period from 1 April to 1 May 2022, totaling 30 days. To unify the time reference and sampling frequency of different sensors, the raw monitoring data are processed into a 1-min interval time series. The model inputs include three-dimensional wind speed, temperature, atmospheric pressure, and humidity, while the outputs are the three-dimensional displacement components at the SHM2 station. The temporal variations of the environmental monitoring variables are shown in Figure 7, and the corresponding three-dimensional dynamic deformation at the SHM2 point is shown in Figure 8. Overall, longitudinal displacement exhibits a relatively large variation range, vertical displacement shows a clear temperature-related variation trend, and lateral displacement has a smaller amplitude and does not show a response pattern dominated by any single environmental variable.

Data preprocessing mainly includes resampling, outlier treatment, time-lag correction, and unit unification. GNSS displacement, three-dimensional wind speed, and meteorological parameters are first resampled to a 1-min interval. A 120-min rolling-window Z-score method is then used to identify outliers exceeding 5σ, and the corresponding positions are filled by temporal interpolation. Considering the time-lag effect between environmental loads and structural responses, cross-correlation analysis is used to determine the optimal lag of meteorological data relative to displacement as 133 min, and temporal alignment is then performed accordingly. Finally, GNSS displacement is converted to millimeters, forming the 9-channel time-series dataset used for subsequent model input.

### 3.3. Multi-Source Data Correlation Analysis and Feature Selection

**Figure 9:**
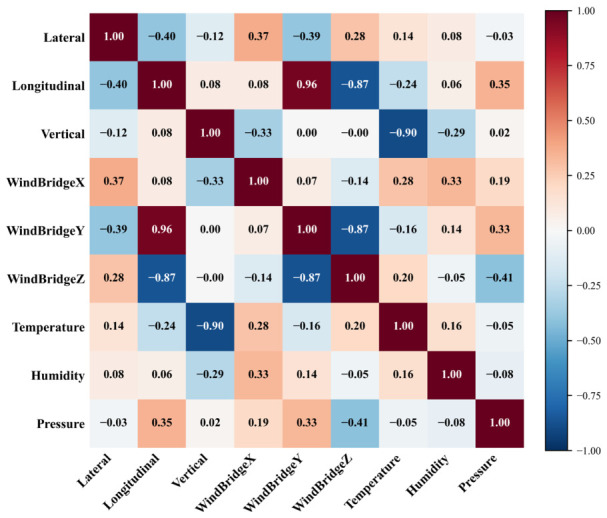
Heatmap of the correlation between multiple loads and three-dimensional deformation at the mid-span of the FRB.

**Figure 10:**
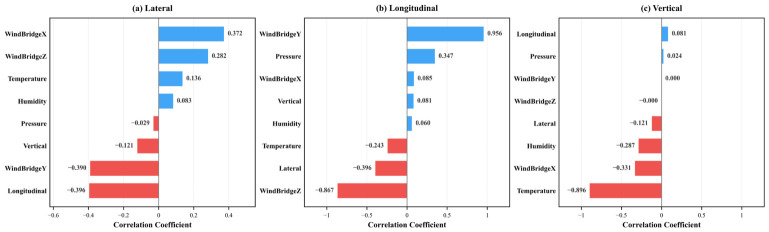
Correlation between multiple loads at the span of the FRB and different sensors.

Correlation among multi-source monitoring variables is an important basis for graph construction and cross-modal information aggregation. Pearson correlation coefficients are used to analyze the associations between environmental loads and three-dimensional displacement. The results are shown in Figure 9 and Figure 10, and the major correlated features are summarized in Table 1. Longitudinal displacement shows the strongest correlations with WindBridgeY and WindBridgeZ, with correlation coefficients of 0.949 and −0.843, respectively. Vertical displacement has a strong correlation with temperature, with a correlation coefficient of −0.780. In contrast, lateral displacement is generally weakly correlated with the current environmental variables, with the maximum correlation coefficient being only −0.302.

The above results indicate that the auxiliary information available for different displacement directions differs substantially. To avoid missing potentially complementary information, all nine variables are included in the model input. The correlation analysis also provides a data basis for the structure of ITimeGAN: the GAT module can construct the graph topology based on feature correlations, while the MACA module uses displacement as Query and environmental variables as Key/Value to characterize the dependency between environmental loads and structural responses. It should be noted that the Pearson correlation coefficient is used only to construct the initial physically interpretable graph topology and does not imply that the model ultimately captures only linear relationships. Subsequent GAT attention weights, cross-modal attention, and the BiLSTM temporal encoder can still adaptively learn nonlinear dynamic relationships across sensors. To avoid an overly dense or sparse graph caused by a fixed threshold, different Pearson threshold settings are further examined during hyperparameter tuning to evaluate their effects on graph sparsity and imputation performance. A higher threshold may remove moderately correlated but physically meaningful edges, leading to insufficient cross-modal information, whereas a lower threshold may introduce weak noisy connections. Considering graph interpretability and imputation accuracy, the final threshold configuration is selected through validation or Hyperband search. Because lateral displacement lacks strongly correlated environmental variables, its imputation performance may be more constrained by insufficient available auxiliary information.

### 3.4. Data Partitioning, Missing Scenarios, and Evaluation Metrics

**Figure 11:**
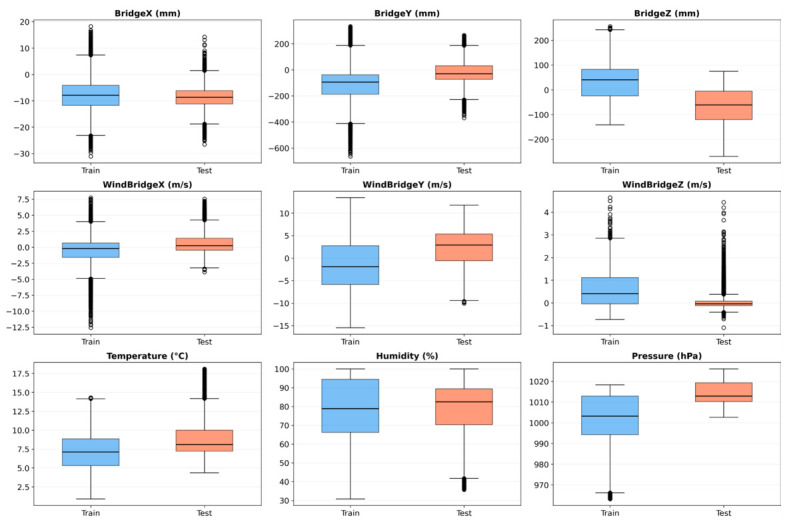
Box plot comparison of feature distributions between training and test sets. The circles in the boxplots indicate outlier points beyond the whiskers.

Considering the continuity of time-series data, the dataset is divided chronologically into training and test sets to avoid future information leakage caused by random shuffling. The data from 1 April to 15 April 2022, are used for model training, while the data from 16 April to 30 April 2022, are used to construct missing scenarios and evaluate imputation performance. The training set is further split internally into training and validation subsets at an 80%/20% ratio for model training, hyperparameter tuning, and early stopping. All features are normalized using MinMaxScaler, with normalization parameters computed only from the training set. The same parameters are used to transform the test set, and the prediction results are inverse normalized before evaluation. As shown in Figure 11, the training and test sets exhibit broadly similar distributions, indicating that the chronological partition does not introduce obvious systematic bias.

**Figure 12:**
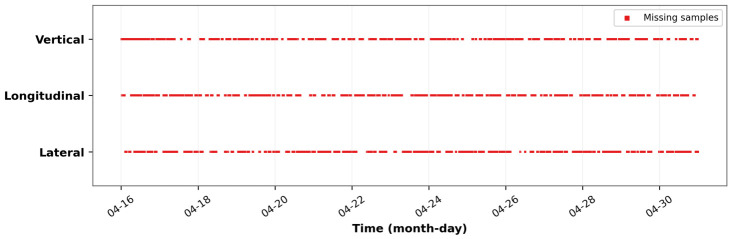
Visualization of random missing masks at five missing rates (30%) for three displacement channels on the test set.

**Figure 13:**
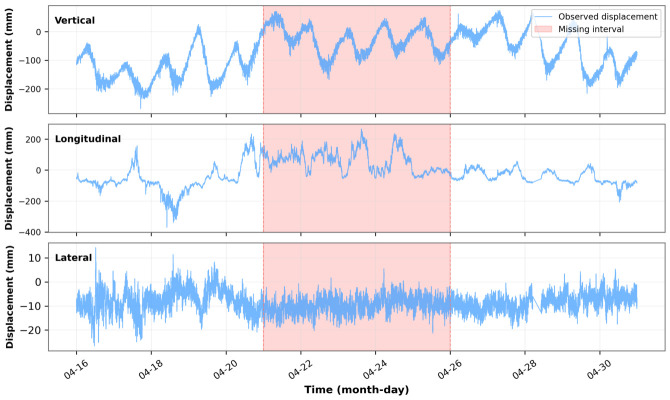
Visualization of continuous missing regions at five durations (5 days) for three displacement channels on the test set, with missing intervals highlighted in red shading.

To evaluate model imputation capability under different missing conditions, two types of missing scenarios are considered: random missing and continuous long-term missing. In the random missing scenario, data points are randomly removed from the target displacement channel, with missing rates set from 10% to 50% and the maximum continuous missing duration not exceeding 60 min. In the continuous long-term missing scenario, continuous segments of 1, 3, 5, 7, and 10 days in the target displacement data are set as completely missing. Both scenarios impose missingness only on the target displacement channel, while environmental variables remain complete. Figure 12 and Figure 13 provide representative mask examples for 30% random missing and 5-day continuous missing, respectively. This setting is used to first evaluate the cross-modal imputation capability under target displacement sensor failure, but it does not yet cover more complex engineering scenarios such as simultaneous failure of multiple displacement channels, partial missingness of environmental variables, or synchronized failures of multiple sensor types.

In both scenarios, the environmental features (wind speed, temperature, humidity, atmospheric pressure) remain intact, with only the target displacement channel containing missing data. This setting is consistent with the common real-world situation of partial sensor failure with remaining sensors operating normally.

Model performance is comprehensively evaluated using the following three metrics:

(17) $$MAE=\frac{1}{m}\sum_{i=1}^{m}|y_{i}-{\hat{y}}_{i}|\;$$

(18) $$RMSE=\sqrt{\frac{1}{m}\sum_{i=1}^{m}(y_{i}-{\hat{y}}_{i}{)}^{2}}$$

(19) $$R^{2}=1-\frac{\sum_{i=1}^{m}(y_{i}-{\hat{y}}_{i}{)}^{2}}{\sum_{i=1}^{m}(y_{i}-\bar{y}{)}^{2}}$$

where $y_{i}$ and ${\hat{y}}_{i\;}$ denote the true value and predicted value of the i sample, respectively, $\;\bar{y}$ is the mean of the true values, and m is the total number of samples. MAE and RMSE measure the absolute error level, and $R^{2}$ measures the model’s ability to explain data variance, with values closer to 1 indicating better fit.

## 4. Experimental Results and Analysis

### 4.1. Experimental Setup and Baseline Models

ITimeGAN (CrossAttn-GNN-TimeGAN) is implemented based on the PyTorch 2.10.0. All experiments are conducted on a workstation equipped with an NVIDIA GeForce RTX 4060 GPU (8 GB memory). Table 2 lists the main training hyperparameters. The model adopts the AdamW optimizer with an initial learning rate of $1\times 10^{-3}$ and a weight decay of $1\times 10^{-4}$. The learning rate schedule follows the ReduceLROnPlateau strategy, halving the learning rate when the validation loss fails to improve for five consecutive epochs. The loss function is defined as an equally weighted sum of MSE and MAE ($L=0.5L_{MSE}+0.5L_{MAE}$), balancing global error minimization and local deviation robustness. The gradient clipping threshold is set to 1.0 to stabilize backpropagation, and an early stopping mechanism (patience = 20 epochs) is employed to prevent overfitting.

To improve the systematic selection of key hyperparameters, this study refers to the Hyperband-based hyperparameter optimization procedure for bridge SHM data restoration. Candidate searches were conducted for the Pearson graph threshold, input sequence length, number of attention heads, and number of GNN layers. Hyperband initially evaluates multiple candidate configurations, successively eliminates poorly performing combinations, and allocates more training resources to better-performing configurations, thereby obtaining favorable parameter settings under limited computational cost. The candidate ranges are listed in Table 2, and the final hyperparameter combinations used for different missing scenarios and displacement directions are summarized in Table 3. The tuning results indicate that the optimal graph threshold is not identical across all targets and missing patterns: some scenarios are better suited to τ = 0.2, which retains more moderately correlated but physically meaningful cross-sensor associations, whereas others are better suited to τ = 0.3, which provides a more stable graph structure. Other hyperparameters, including seq_len, nhead, and gnn_layers, were selected through the same Hyperband procedure.

As shown in Table 3, longitudinal and lateral displacement under random missing are better suited to a lower graph threshold of τ = 0.2, whereas vertical displacement and continuous missing scenarios are better suited to τ = 0.3. This difference is related to the physical correlation characteristics of different displacement directions and the information availability under different missing patterns. It indicates that graph structure and attention configuration should be selected according to validation performance rather than fixed as a single parameter set for all scenarios.

Five baseline models are compared in this study: TimeGAN (an ablation baseline without GAT/MACA), VAE (Variational Autoencoder), AE (Autoencoder), STGCN (Spatiotemporal Graph Convolutional Network), and BDLM (Bayesian Dynamic Linear Model). The ITimeGAN results are obtained using the final hyperparameter combinations listed in Table 4. All models use the same data partition, preprocessing workflow, and evaluation procedure. Under the same computational environment, using the random 30% pctscenario and BridgeX target as a representative case, ITimeGAN has 1,257,153 trainable parameters; point-wise imputation over the full test windows takes approximately 54.97 s in total, corresponding to about 2.55 ms per point. This inference cost is small relative to the minute-level sampling interval commonly used in bridge SHM and is suitable for periodic data quality control and offline missing-data recovery.

### 4.2. Random Missing Scenario Imputation Results

**Figure 14:**
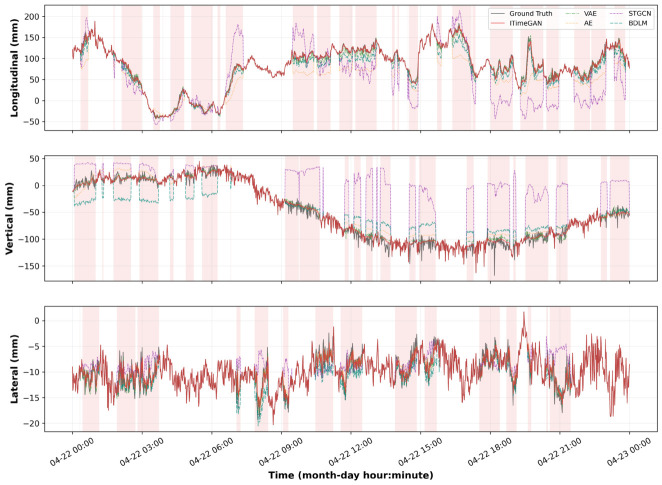
Zoomed-in imputation waveform comparison for three displacement directions under 50% random missing.

Table 5 summarizes the representative imputation performance of ITimeGAN and external baseline models under 30% random missing for the three displacement directions. The ITimeGAN results use the final hyperparameter combinations listed in Table 3 for the corresponding scenarios and displacement directions. Supplementary results for the other random missing rates (10%, 20%, 40%, and 50%) are provided in Appendix A Table A1. Figure 14 shows local waveform comparisons for the three displacement directions under 50% random missing.

For longitudinal displacement, ITimeGAN shows stable imputation performance under random missing scenarios, with R^2^ values above 0.9944 and MAE values between 3.28 and 4.26 mm across missing rates of 10–50%. At 30% missing, ITimeGAN achieves MAE = 4.26 mm and R^2^ = 0.9944, outperforming AE and STGCN and remaining comparable to or better than VAE and BDLM. This indicates that when strong physical associations exist between the target response and environmental loads, graph-based correlation encoding and cross-modal information aggregation can effectively exploit auxiliary variables such as wind speed. By contrast, STGCN obtains R^2^ values of only 0.6180–0.6488 for longitudinal displacement, suggesting that conventional spatiotemporal graph convolution does not adequately represent rapid wind-driven displacement responses in this small-scale heterogeneous sensor graph. Although BDLM performs better than STGCN, its linear dynamic assumption still limits its ability to describe complex nonlinear variations.

The vertical displacement results are more scenario-dependent. ITimeGAN obtains R^2^ values between 0.9073 and 0.9682 under random missing, but it does not outperform TimeGAN or AE at some missing rates. This suggests that vertical displacement is mainly governed by temperature-induced periodic and slowly varying thermal deformation, and that a denser graph structure or more complex cross-feature aggregation does not always lead to monotonic improvement. VAE gradually degrades as the missing rate increases, with R^2^ decreasing from 0.8630 at 10% missing to 0.7967 at 50% missing, indicating limited ability to preserve the amplitude and phase of periodic responses. STGCN and BDLM produce relatively large errors; in particular, the negative R^2^ values of STGCN indicate that simple graph convolution may be unstable in low-dimensional heterogeneous sensor systems if the role difference between loads and responses is not explicitly modeled.

Lateral displacement is challenging for all models. ITimeGAN achieves relatively stable but limited results under random missing, with R^2^ values of approximately 0.7562–0.7911 and MAE values of 1.41–1.46 mm. This is consistent with the correlation analysis in Section 3.2: lateral displacement lacks strong correlations with the available environmental variables, limiting the amount of usable cross-modal information. Hyperband tuning helps retain some moderately correlated edges and improves the use of self-history and weakly correlated environmental information. However, in the absence of traffic loads, multi-station displacement, or more direct lateral load information, the improvement is still constrained by the available observations. The weaker performance of STGCN and BDLM for lateral displacement further indicates that weakly correlated targets cannot be reliably recovered using conventional graph convolution or linear dynamic models alone.

In terms of sensitivity to missing rate, ITimeGAN does not show obvious degradation for longitudinal and lateral displacement as the missing rate increases, indicating that the validated parameter configurations maintain stable recovery capability under random missing. The vertical displacement results suggest that hyperparameter tuning does not provide consistent gains for all targets, and model configuration should be selected according to the physical correlation of the target response and validation performance. Overall, the results in Table 5 and Appendix A Table A1 support the proposed collaborative imputation design, while also showing that recoverability differs substantially across displacement directions depending on the quality of available auxiliary information.

### 4.3. Continuous Long-Term Missing Scenario Imputation Results

**Figure 15:**
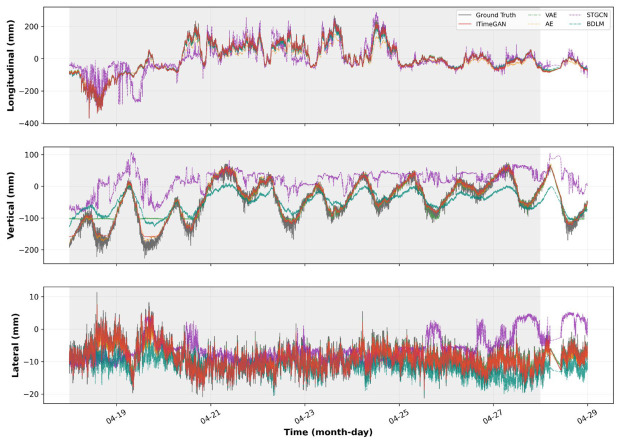
Imputation waveform comparison for three displacement directions under 10-day continuous missing.

**Figure 16:**
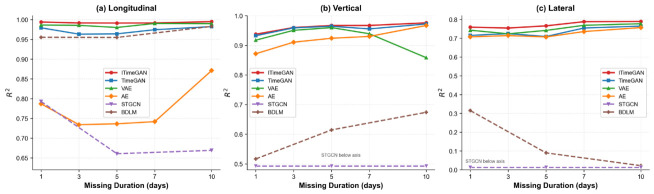
Effect of continuous missing duration on R^2^ for each model.

The continuous long-term missing scenario represents a more rigorous test for imputation methods—the model must rely entirely on cross-modal information from environmental features for long-duration reconstruction without any ground-truth anchor points from the target channel. Table 6 summarizes the representative imputation metrics of ITimeGAN and external baseline models under 5-day continuous missing. Supplementary results for the other continuous missing durations (1, 3, 7, and 10 days) are provided in Appendix A Table A2. Figure 15 shows waveform comparisons for the three displacement directions under 10-day continuous missing, with the missing intervals shaded in gray; Figure 16 further shows the effect of missing duration on R^2^ for different models.

In continuous missing scenarios, ITimeGAN uses the final hyperparameter combinations listed in Table 6 to prioritize stability during long-horizon prediction. Taking 5-day continuous missing as an example, ITimeGAN achieves R^2^ 0.9913 and MAE 4.58 mm for longitudinal displacement, outperforming VAE, AE, STGCN, and BDLM. These results indicate that when the target channel has no observation anchor for a long duration, explicitly using the correlations between environmental loads and displacement is particularly important for stable structural response reconstruction.

For vertical displacement under continuous missing, both ITimeGAN and TimeGAN maintain high R^2^ values, with ITimeGAN slightly outperforming TimeGAN for most missing durations. ITimeGAN increases from R^2^ 0.9374 at 1-day missing to R^2^ 0.9759 at 10-day missing, while TimeGAN increases from 0.9324 to 0.9721; the gap between them is generally small. VAE degrades significantly for long durations, with R^2^ decreasing to 0.8582 and MAE increasing to 13.58 mm at 10 days. STGCN and BDLM show higher errors, indicating that although vertical displacement is strongly correlated with temperature, preserving the phase and amplitude of its periodic variation still requires both temporal modeling and cross-modal information utilization.

Lateral displacement R^2^ remains lower than for the other two directions under continuous missing, and most models have MAE values around 1.40–1.60 mm or higher. ITimeGAN is generally better than STGCN and BDLM, but its advantage over TimeGAN, VAE, and AE is not very pronounced. This suggests that the main difficulty of lateral displacement imputation is not only model structure, but also the lack of auxiliary information strongly correlated with lateral response in the current sensor configuration. Under limited observed variables, the model can maintain a certain degree of stability, but further improvement would require traffic loads, multi-station displacement, local lateral wind fields, or other monitoring variables more directly related to lateral response.

### 4.4. Ablation Analysis and Module Contributions

**Figure 17:**
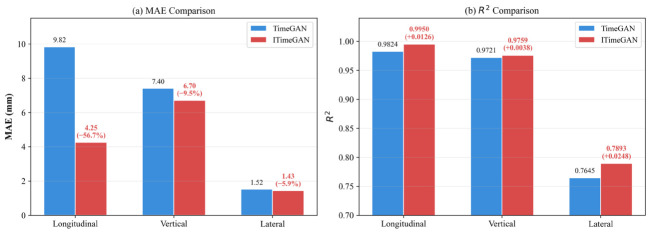
Ablation analysis: MAE comparison between ITimeGAN and TimeGAN under 10-day continuous missing.

To quantify the contribution of the core modules in ITimeGAN, this study directly compares ITimeGAN (CrossAttn-GNN-TimeGAN) with the TimeGAN baseline. The main difference is that ITimeGAN additionally introduces GAT graph attention encoding and the cross-modal attention module, and increases the input sequence length from 60 to 90 to accommodate longer temporal context. Table 7 selects two representative scenarios, 30% random missing and 10-day continuous missing, to compare performance differences and improvements for the three displacement directions; supplementary ablation results under 50% random missing are provided in Appendix A Table A3. Figure 17 shows the grouped MAE bars of ITimeGAN and TimeGAN for the three displacement directions under 10-day continuous missing.

The ablation analysis shows three main trends. First, under continuous long-term missing scenarios, ITimeGAN outperforms TimeGAN for all three targets, with the largest improvement for longitudinal displacement. Its MAE decreases from 9.82 mm to 4.25 mm, corresponding to a reduction of approximately 57%. This indicates that GAT and cross-modal attention make important contributions when the target channel has no long-term observations. GAT encodes the strong association between wind speed and displacement into graph representations, while cross-modal attention uses displacement as Query and environmental loads as Key/Value to aggregate environmental driving information, thereby improving longitudinal displacement reconstruction.

Second, in random missing scenarios, after Hyperband parameter selection, ITimeGAN shows clearer advantages for longitudinal and lateral displacement. At 30% random missing, for example, the R^2^ of longitudinal displacement increases from 0.9806 for TimeGAN to 0.9944, and MAE decreases from 8.39 mm to 4.26 mm. For lateral displacement, R^2^ increases from 0.7092 to 0.7686. These results indicate that a lower Pearson threshold and deeper graph aggregation can help retain moderately strong cross-sensor associations in some random missing scenarios, improving graph encoding. However, for vertical displacement, the original parameters are more robust, showing that hyperparameter optimization does not produce monotonic gains for all targets.

Third, the improvement for lateral displacement remains limited, with MAE reductions below 0.2 mm. This is consistent with the correlation analysis in Section 3.2: when there is no strong correlation between the target and environmental variables, the effective physical information available to GAT and cross-modal attention remains limited even after adjusting graph thresholds and attention structures. Thus, module contribution is constrained by the information provided by the sensor system.

### 4.5. Discussion

Based on the comprehensive experimental results above, the following analysis and discussion are presented:

Consistency between model performance ranking and physical correlations: The imputation difficulty of the three displacement directions follows the order longitudinal displacement (easiest) > vertical displacement (moderate) > lateral displacement (most difficult). This order is consistent with the correlation strengths revealed in Section 3.2: longitudinal displacement has a strong correlation with WindBridgeY (0.949), vertical displacement is strongly correlated with temperature (−0.780), and lateral displacement has only weak correlations with the available variables, with the maximum coefficient being −0.302. This indicates that, for cross-modal imputation methods, the quality of available auxiliary information, namely the physical association between environmental loads and target responses, is a key factor determining the achievable performance. From the imputed waveforms, the reconstructed longitudinal displacement remains consistent with wind-dominated response variations, while the reconstructed vertical displacement preserves slow-varying and periodic features associated with temperature changes, indicating that the imputation results are qualitatively consistent with basic bridge-response behavior.

Difficulty difference between random and continuous missing: Continuous long-term missing is intuitively more challenging than random missing because the target channel loses observation anchors for an extended period. However, the experimental results show that the difficulty is also affected by physical correlation and parameter configuration. For longitudinal displacement, ITimeGAN achieves R^2^ = 0.9950 under 10-day continuous missing and R^2^ = 0.9944 under 30% random missing. Both values remain high, indicating that when environmental driving information is sufficiently strong, the model can reconstruct the target response stably from complete environmental variables. For vertical and lateral displacement, performance is more constrained by temperature periodicity and weak auxiliary information.

Imputation bottleneck for weakly correlated targets: Lateral displacement shows lower imputation accuracy than the other two directions across models and scenarios, with the highest R^2^ around 0.79. This bottleneck does not solely reflect insufficient model capacity, but also the intrinsic information limitation of the current sensor configuration. Lateral displacement may be influenced by unobserved factors such as traffic loads, lane distribution, bridge geometric asymmetry, local boundary conditions, and spatial differences in lateral wind fields. Therefore, improving lateral displacement imputation requires additional monitoring variables more directly related to lateral response, such as traffic flow, vehicle load, multi-station GNSS displacement, or denser wind-field observations.

Positioning relative to recent methods: Compared with general time-series imputation models such as SAITS [21], the proposed method does not only model temporal dependencies and cross-feature correlations, but also explicitly distinguishes the roles of structural responses and environmental driving variables. Compared with graph attention-based imputation methods such as ST D-GAT [25], the target of this study is not a large-scale remote-sensing deformation field, but a low-dimensional multi-source heterogeneous bridge sensor system. Overall, the proposed framework combines a feature correlation graph, load–response cross-modal attention, and a temporal encoder–decoder to address collaborative reconstruction under long-term missingness of the target displacement channel.

Scope and limitations: The current validation is based on one bridge, one monitoring month, and a limited sensor configuration, and therefore does not fully demonstrate generalization across bridge types, seasons, or extreme environments. The missing scenarios also assume complete environmental variables and missingness only in the target displacement channel; simultaneous multi-sensor failures, partial missingness of environmental variables, and online inference efficiency have not yet been systematically evaluated. Future work should include multi-bridge and multi-station datasets, traffic load information, compound missing scenarios, computational efficiency assessment, multi-seed stability analysis, and cross-dataset generalization tests.

## 5. Conclusions

To address the problem of multi-source heterogeneous data missing in bridge structural health monitoring, this paper proposes a missing data imputation method (ITimeGAN) that integrates a GAT, cross-modal attention mechanism, and temporal generative adversarial framework, and systematically validates it on the measured monitoring data from the GeoSHM system of the Forth Road Bridge. The main conclusions are as follows:

1. ITimeGAN achieves excellent imputation performance under both random missing and continuous long-term missing scenarios. Under random missing, $R^{2}$ for longitudinal displacement exceeds 0.99 at most missing rates, and $R^{2}$ for vertical displacement is maintained in the 0.91–0.97 range. Under the extreme scenario of 10-day continuous missing, $R^{2}$ for longitudinal displacement reaches 0.9950 (MAE = 4.25 mm) and $R^{2}$ for vertical displacement reaches 0.9759 (MAE = 6.70 mm), demonstrating reliable reconstruction capability under prolonged absence of target-channel observations.
2. Ablation experiments demonstrate that the joint introduction of the GAT graph attention encoding module and the cross-modal attention mechanism contributes significantly to targets with strong environmental correlations (longitudinal displacement, vertical displacement). Under 10-day continuous missing, ITimeGAN reduces the MAE of longitudinal displacement by 57% compared with the TimeGAN baseline, from 9.82 mm to 4.25 mm. This supports the effectiveness of combining physically related graph encoding with environmental–information cross-modal aggregation.
3. The imputation performance differences across three displacement directions are strictly consistent with the physical correlation strengths revealed by Pearson correlation analysis—longitudinal displacement (wind speed correlated, r=0.949) performs best, vertical displacement (temperature correlated, r=−0.780) is second, and lateral displacement (weakly correlated, r≤0.302) is the most challenging. This finding indicates that for cross-modal information-based data imputation methods, the performance upper bound depends on the strength of the physical association between the target response and the available auxiliary information.
4. ITimeGAN has practical engineering application value. The method can reconstruct structural displacement responses during long-term target-channel failure using environmental sensor data, providing a feasible approach for maintaining data integrity in bridge SHM systems during prolonged sensor malfunction.

This study has the following limitations that warrant improvement in future work: (a) The imputation accuracy of lateral displacement is constrained by the lack of auxiliary information strongly correlated with transverse displacement in the current monitoring configuration; future work may incorporate traffic-flow data or multi-station displacement information to improve lateral response reconstruction. (b) The current missing scenarios assume complete environmental variables and missingness only in the target displacement channel. More complex engineering scenarios, such as partial missingness of environmental variables, simultaneous missingness of multiple displacement channels, or synchronized failures of multiple sensor types, have not yet been covered. Future work will construct more realistic compound missing scenarios to evaluate engineering applicability under multi-source monitoring system failures. (c) The current missing scenarios assume complete environmental variables and missingness only in the target displacement channel. More complex engineering scenarios, such as partial missingness of environmental variables, simultaneous missingness of multiple displacement channels, or synchronized failures of multiple sensor types, have not yet been covered. Future work will construct more realistic compound missing scenarios to evaluate engineering applicability under multi-source monitoring system failures. (d) The current experiments have not systematically evaluated multiple random seeds or repeated random missing masks, and the efficiency assessment is still limited to a representative single-GPU setting. Future work will supplement statistical stability and system-level online efficiency analyses and explore online incremental learning strategies to enable model parameters to adapt to streaming monitoring data.

## Figures and Tables

**Table 1:** Correlation coefficients between displacements and multi-load factors.

Target	First-Correlated Feature	Second	Third
Lateral displacement	WindBridgeY (−0.302)	DisplacementY (−0.272)	WindBridgeZ (0.228)
Longitudinal displacement	WindBridgeY (0.949)	WindBridgeZ (−0.843)	Pressure (0.401)
Vertical displacement	Temperature (−0.780)	WindBridgeX (−0.405)	Pressure (−0.238)

**Table 2:** Search range of key ITimeGAN hyperparameters.

Hyperparameter	Symbol	Search Range
Pearson graph threshold	τ	0.2, 0.3, 0.4, 0.5
Input sequence length	seq_len	60, 90, 120
Number of attention heads	nhead	2, 4, 8
Number of GNN layers	gnn_layers	1, 2, 3

**Table 3:** Final ITimeGAN parameters adopted under different missing scenarios and displacement directions.

Missing Type	Target Displacement	τ	seq_len	nhead	gnn_layers
Random missing	Longitudinal displacement	0.2	90	4	3
	Vertical displacement	0.3	90	4	2
	Lateral displacement	0.2	90	2	3
Continuous missing	Longitudinal displacement	0.3	90	4	2
	Vertical displacement	0.3	90	4	2
	Lateral displacement	0.3	90	4	2

**Table 4:** Main training hyperparameters.

Parameter	ITimeGAN	TimeGAN	VAE	AE	STGCN	BDLM
Input sequence length	90	60	60	60	90	90
Batch size	64	64	64	64	64	—
Maximum epochs	100	100	100	100	100	—
Initial learning rate	1 × 10^−3^	1 × 10^−3^	1 × 10^−3^	1 × 10^−3^	1 × 10^−3^	—
Optimizer	AdamW	AdamW	AdamW	AdamW	AdamW	Kalman update
GAT/GCN hidden dimension	64	—	—	—	64	—
Temporal encoder hidden dimension	128	128	128	128	64	—
Early stopping patience	20	20	20	20	20	—

**Table 5:** Imputation performance under 30% random missing (MAE/RMSE: mm; R^2^: dimensionless).

Target	Model	MAE	RMSE	R^2^
Longitudinal displacement	ITimeGAN	4.26	6.36	0.9944
	VAE	4.59	7.04	0.9931
	AE	16.99	22.30	0.9310
	STGCN	37.90	52.46	0.6180
	BDLM	7.98	11.14	0.9828
Vertical displacement	ITimeGAN	11.41	18.34	0.9279
	VAE	16.46	27.00	0.8438
	AE	10.37	13.63	0.9602
	STGCN	64.34	77.77	−0.2964
	BDLM	28.79	34.04	0.7516
Lateral displacement	ITimeGAN	1.41	1.83	0.7686
	VAE	1.49	1.93	0.7416
	AE	1.57	2.06	0.7069
	STGCN	5.15	6.57	−1.9886
	BDLM	3.13	3.83	−0.0160

**Table 6:** Imputation performance under 5-Day continuous missing (MAE/RMSE: mm; R^2^: dimensionless).

Target	Model	MAE	RMSE	R^2^
Longitudinal displacement	ITimeGAN	4.58	6.47	0.9913
	VAE	7.24	9.76	0.9802
	AE	29.37	35.61	0.7363
	STGCN	31.47	40.40	0.6606
	BDLM	11.14	14.72	0.9549
Vertical displacement	ITimeGAN	5.93	8.12	0.9669
	VAE	6.58	8.95	0.9598
	AE	9.48	12.33	0.9238
	STGCN	53.92	65.37	−1.1438
	BDLM	23.68	27.72	0.6145
Lateral displacement	ITimeGAN	1.42	1.84	0.7659
	VAE	1.49	1.93	0.7416
	AE	1.57	2.06	0.7069
	STGCN	3.46	4.40	−0.9173
	BDLM	2.45	3.03	0.0897

**Table 7:** Ablation analysis: Comparison between the proposed method and TimeGAN Baseline (MAE: mm; R^2^: dimensionless).

Scenario	Component	ITimeGAN R^2^	TimeGAN R^2^	R^2^ Improvement	ITimeGAN MAE	TimeGAN MAE	MAE Reduction	MAE Reduction Rate
Random 30%	Longitudinal displacement	0.9944	0.9806	+0.0138	4.26	8.39	−4.13	49.2%
	Vertical displacement	0.9279	0.9629	−0.0350	11.41	9.26	+2.16	−23.2%
	Lateral displacement	0.7686	0.7092	+0.0594	1.41	1.58	−0.17	10.8%
Continuous 10 days	Longitudinal displacement	0.9950	0.9824	+0.0126	4.25	9.82	−5.57	56.7%
	Vertical displacement	0.9759	0.9721	+0.0038	6.70	7.40	−0.70	9.5%
	Lateral displacement	0.7893	0.7645	+0.0248	1.43	1.52	−0.09	5.9%

## Data Availability

The data presented in this study are available on request from the corresponding author. Due to contractual restrictions imposed by the data owner (the UK project partner of the ESA-funded GeoSHM project), the dataset cannot be made publicly available.

## Appendix A. Supplementary Experimental Results

**Table A1:** Random Missing (10%, 20%, 40%, 50%).

Target	Missing Rate	Model	MAE	RMSE	R^2^
Longitudinal displacement	10%	ITimeGAN	3.63	5.45	0.9957
		VAE	5.37	7.57	0.9918
		AE	18.59	25.18	0.9092
		STGCN	35.65	49.51	0.6488
		BDLM	8.14	11.05	0.9825
	20%	ITimeGAN	3.77	6.00	0.9949
		VAE	4.34	6.15	0.9947
		AE	23.81	33.21	0.8444
	40%	ITimeGAN	3.70	5.75	0.9954
		VAE	4.27	6.22	0.9946
		AE	25.89	36.64	0.8125
	50%	ITimeGAN	3.28	5.34	0.9962
		VAE	4.28	6.39	0.9945
		AE	19.61	25.94	0.9097
		STGCN	37.70	52.03	0.6368
		BDLM	7.84	10.91	0.9840
Vertical displacement	10%	ITimeGAN	10.71	19.20	0.9073
		VAE	13.95	23.34	0.8630
		AE	19.27	23.12	0.8656
		STGCN	60.41	72.80	−0.3326
		BDLM	24.99	31.16	0.7558
	20%	ITimeGAN	7.40	11.75	0.9682
		VAE	15.01	24.45	0.8623
		AE	9.52	12.60	0.9634
	40%	ITimeGAN	10.74	17.10	0.9362
		VAE	17.95	29.21	0.8139
		AE	12.92	16.67	0.9394
	50%	ITimeGAN	9.75	14.83	0.9542
		VAE	19.03	31.23	0.7967
		AE	14.38	18.35	0.9298
		STGCN	69.26	83.20	−0.4428
		BDLM	30.29	36.00	0.7298
Lateral displacement	10%	ITimeGAN	1.46	1.89	0.7562
		VAE	1.51	1.94	0.7427
		AE	1.60	2.07	0.7072
		STGCN	4.78	5.99	−1.4515
		BDLM	2.92	3.69	0.0678
	20%	ITimeGAN	1.42	1.84	0.7584
		VAE	1.52	1.97	0.7235
		AE	1.54	2.00	0.7142
	40%	ITimeGAN	1.44	1.88	0.7875
		VAE	1.51	1.96	0.7688
		AE	1.61	2.10	0.7355
	50%	ITimeGAN	1.43	1.85	0.7911
		VAE	1.48	1.92	0.7771
		AE	1.55	2.00	0.7567
		STGCN	4.99	6.39	−1.4780
		BDLM	3.13	3.85	0.1000

**Table A2:** Continuous Missing (1, 3, 7, 10 Days).

Target	Duration	Model	MAE	RMSE	R^2^
Longitudinal displacement	1 day	ITimeGAN	4.85	6.70	0.9940
		VAE	7.39	10.05	0.9864
		AE	32.24	39.78	0.7870
		STGCN	30.60	39.24	0.7928
		BDLM	13.98	18.22	0.9553
	3 days	ITimeGAN	4.94	6.74	0.9916
		VAE	6.58	8.89	0.9855
		AE	29.92	38.05	0.7341
	7 days	ITimeGAN	4.84	6.86	0.9915
		VAE	5.38	7.31	0.9903
		AE	28.20	37.71	0.7418
	10 days	ITimeGAN	4.25	6.68	0.9950
		VAE	6.43	9.51	0.9899
		AE	25.19	34.00	0.8712
		STGCN	38.11	54.49	0.6691
		BDLM	8.91	12.38	0.9829
Vertical displacement	1 day	ITimeGAN	5.33	7.46	0.9374
		VAE	6.63	8.58	0.9173
		AE	8.26	10.69	0.8715
		STGCN	63.57	73.47	−5.0736
		BDLM	17.57	20.73	0.5164
	3 days	ITimeGAN	5.98	8.46	0.9600
		VAE	6.75	9.40	0.9506
		AE	9.84	12.64	0.9106
	7 days	ITimeGAN	6.33	8.47	0.9670
		VAE	8.07	11.52	0.9390
		AE	9.84	12.34	0.9300
	10 days	ITimeGAN	6.70	9.64	0.9759
		VAE	13.58	23.38	0.8582
		AE	8.65	11.30	0.9669
		STGCN	65.97	80.19	−0.6673
		BDLM	29.65	35.47	0.6739
Lateral displacement	1 day	ITimeGAN	1.45	1.88	0.7589
		VAE	1.51	1.94	0.7427
		AE	1.60	2.07	0.7072
		STGCN	3.38	4.05	−0.9382
		BDLM	1.94	2.41	0.3150
	3 days	ITimeGAN	1.43	1.85	0.7543
		VAE	1.52	1.97	0.7235
		AE	1.54	2.00	0.7142
	7 days	ITimeGAN	1.44	1.88	0.7882
		VAE	1.51	1.96	0.7688
		AE	1.61	2.10	0.7355
	10 days	ITimeGAN	1.43	1.86	0.7893
		VAE	1.48	1.92	0.7771
		AE	1.55	2.00	0.7567
		STGCN	4.50	5.93	−1.2798
		BDLM	3.15	3.88	0.0218

**Table A3:** ITimeGAN–TimeGAN Ablation Comparison.

Missing Type	Component	Scenario	ITimeGAN R^2^	TimeGAN R^2^	R^2^ Improvement	ITimeGAN MAE	TimeGAN MAE	MAE Reduction Rate
Random	Longitudinal displacement	10%	0.9957	0.9869	+0.0088	3.63	7.21	49.7%
		20%	0.9949	0.9808	+0.0141	3.77	7.45	49.4%
		30%	0.9944	0.9806	+0.0138	4.26	8.39	49.2%
		40%	0.9954	0.9916	+0.0038	3.70	5.82	36.4%
		50%	0.9962	0.9670	+0.0292	3.28	10.89	69.9%
	Lateral displacement	10%	0.7562	0.7151	+0.0411	1.46	1.59	8.2%
		20%	0.7584	0.7245	+0.0339	1.42	1.51	6.0%
		30%	0.7686	0.7092	+0.0594	1.41	1.58	10.8%
		40%	0.7875	0.7544	+0.0331	1.44	1.55	7.1%
		50%	0.7911	0.7645	+0.0266	1.43	1.52	5.9%
Continuous	Longitudinal displacement	1 day	0.9940	0.9793	+0.0147	4.85	9.86	50.8%
		3 days	0.9916	0.9633	+0.0283	4.94	10.73	54.0%
		5 days	0.9913	0.9640	+0.0273	4.58	10.74	57.4%
		7 days	0.9915	0.9745	+0.0170	4.84	8.47	42.9%
		10 days	0.9950	0.9824	+0.0126	4.25	9.82	56.7%
	Vertical displacement	1 day	0.9374	0.9324	+0.0050	5.33	5.44	2.0%
		3 days	0.9600	0.9589	+0.0011	5.98	6.22	3.9%
		5 days	0.9669	0.9643	+0.0026	5.93	6.43	7.8%
		7 days	0.9670	0.9554	+0.0116	6.33	6.97	9.2%
		10 days	0.9759	0.9721	+0.0038	6.70	7.40	9.5%
	Lateral displacement	1 day	0.7589	0.7151	+0.0438	1.45	1.59	8.8%
		3 days	0.7543	0.7245	+0.0298	1.43	1.51	5.3%
		5 days	0.7659	0.7092	+0.0567	1.42	1.58	10.1%
		7 days	0.7882	0.7544	+0.0338	1.44	1.55	7.1%
		10 days	0.7893	0.7645	+0.0248	1.43	1.52	5.9%
